# [^18^F]-Fluoro-Deoxy-Glucose Positron Emission Tomography Scan Should Be Obtained Early in Cases of Autoimmune Encephalitis

**DOI:** 10.1155/2016/9450452

**Published:** 2016-07-31

**Authors:** C. R. Newey, A. Sarwal, S. Hantus

**Affiliations:** ^1^Department of Neurology, University of Missouri, 5 Hospital Drive, CE 540, Columbia, MO 65211, USA; ^2^Neurology and Critical Care, Wake Forest University School of Medicine, Reynolds M, Medical Center Boulevard, Winston-Salem, NC 27157, USA; ^3^Department of Adult Neurology, Cleveland Clinic, 9500 Euclid Avenue, Cleveland, OH 44195-5245, USA; ^4^Epilepsy Center, Neurologic Institute, Cleveland Clinic, 9500 Euclid Avenue, Cleveland, OH 44195-5245, USA

## Abstract

*Introduction*. Autoimmune encephalitis (AE) is a clinically challenging diagnosis with nonspecific neurological symptoms. Prompt diagnosis is important and often relies on neuroimaging. We present a case series of AE highlighting the importance of an early [^18^F]-fluoro-deoxy-glucose positron emission tomography (FDG-PET) scan.* Methods*. Retrospective review of seven consecutive cases of autoimmune encephalitis.* Results*. All patients had both magnetic resonance imaging (MRI) and FDG-PET scans. Initial clinical presentations included altered mental status and/or new onset seizures. Six cases had serum voltage-gated potassium channel (VGKC) antibody and one had serum N-methyl-D-aspartate (NMDA) antibody. MRI of brain showed mesial temporal lobe hyperintensity in five cases of VGKC. The other two patients with VGKC or NMDA AE had restiform body hyperintensity on MRI brain or a normal MRI, respectively. Mesial temporal lobe hypermetabolism was noted in three cases on FDG-PET, despite initial unremarkable MRI. Malignancy workup was negative in all patients.* Conclusion*. A high index of suspicion for AE should be maintained in patients presenting with cognitive symptoms, seizures, and limbic changes on neuroimaging. In cases with normal initial brain MRI, FDG-PET can be positive. Additionally, extralimbic hyperintensity on MRI may also be observed.

## 1. Introduction

Autoimmune encephalitis (AE) is a clinically challenging diagnosis usually suspected in cases of altered mental status or seizures after excluding other more common diagnoses. (Anderson 2008) Common presenting signs are nonspecific-memory impairment, hallucinations, anxiety, irritability, depression, seizures, or sleep alterations [1, 2]. These symptoms may develop over a course of weeks to months. AE may be of infectious, paraneoplastic, or immune-mediated etiology. Differential diagnoses, diagnostic approaches, and clinical criteria for diagnosis of paraneoplastic AE have been proposed previously [3–5]. Most recently, consensus on diagnostic criteria for autoimmune encephalitis has been published [6]. Positive serology with specific antibodies and negative workup for malignancy are important in the diagnosis [6]. With emerging evidence of reversibility of AE with immunotherapy, early diagnosis is important and should not be delayed while awaiting for serological data [6]. Thus, early neuroimaging, along with the initial neurological assessment, plays an important role [6, 7].

Voltage-gated potassium channel (VGKC) and N-methyl-D-aspartate (NMDA) antibodies are increasingly being recognized as associations of AE due to easy availability of serological testing. Since the presenting symptoms are nonspecific, establishing neuroimaging correlates to guide diagnostic workup may allow for timely therapeutic interventions. We present seven patients (six positive for VGKC and one positive for NMDA antibodies) with AE highlighting the importance of early neuroimaging with brain magnetic resonance imaging (MRI) and [^18^F]-fluoro-deoxy-glucose positron emission tomography (FDG-PET).

## 2. Methods

We screened medical records of patients evaluated at a tertiary academic medical center between 2004 and 2010 billed under ICD-9 codes 276.1 and 323.9. A retrospective review of 848 case records with clinical diagnosis of AE and/or limbic encephalitis upon presentation was done. Of these, 102 patients had paraneoplastic panel results available with 7 being positive (6 positive for VGK and 1 positive for NMDA antibodies). All panels were processed by Mayo Medical Laboratories (Rochester, MN). The cases were further reviewed for historical information, diagnostic workup, medications administered, and neuroimaging findings. The Institutional Review Board approved the study protocol.

## 3. Results

Patient characteristics are described in Table 1. Mean age was 56.7 years (range: 22 to 91 years of age; 4 males and 3 females). All presented with the chief complaint of altered mental status and/or seizure. One patient had elevated microsomal antibodies (138.6 IU/mL; normal < 5 IU/mL) and thyroglobulin antibodies (1209 IU/mL; normal < 10 IU/mL) with a normal thyroid stimulating hormone (TSH) (4.63 *μ*U/mL) and free T4 (1.4 *μ*U/mL) in addition to VGKC antibody. None of the patients had diabetes. Computed tomography (CT) of chest, abdomen, and pelvis was negative in all patients. CSF was significant in three patients with elevation of white cell count in two patients and mild elevation of protein in a third patient.

The mean time of symptom onset to positive antibody serology was 102.1 days (range: 8 to 185 days; Table 1). The average VGKC titer on initial presentation was 1.44 nmol/L (normal < 0.03) [8]. NMDA titer was not resulted. All but one patient received intravenous methylprednisolone, intravenous immunoglobulin, and plasmapheresis in various combinations. Four of the six treated patients improved with treatment with minimal residual cognitive deficits. One patient did not receive treatment and improved remarkably prior to discharge and returned to her baseline health five months after presentation. One patient expired from an unrelated medical cause. One patient progressed to persistent vegetative state. At least three patients had serial serology titers for the antibodies. Only one case made significant improvement in titer level following treatment. Six of the seven patients required prolonged antiepileptic agents for seizure control.

The average time between symptom onset and first neuroimaging modality was 48.6 days (range: 4 to 184 days). MRI of the brain was the first modality in four cases (Table 2). CT of head was performed first in the other cases. The average time from onset of symptoms to first positive finding on neuroimaging (i.e., T2/fluid attenuated inversion recovery (FLAIR) hyperintensity on MRI or FDG-PET hypermetabolism) was 80.0 days (range: 16 to 184 days). Four patients had mesial temporal lobe hyperintensity on T2/FLAIR weighted MRI. One patient with VGKC AE had T2/FLAIR weighted hyperintensity of the restiform bodies. The other patient with VGKC AE had global atrophy. The patient with positive NMDA AE had serial MRIs that were all unremarkable except development of generalized atrophy (Table 3).

**Figure 1:**
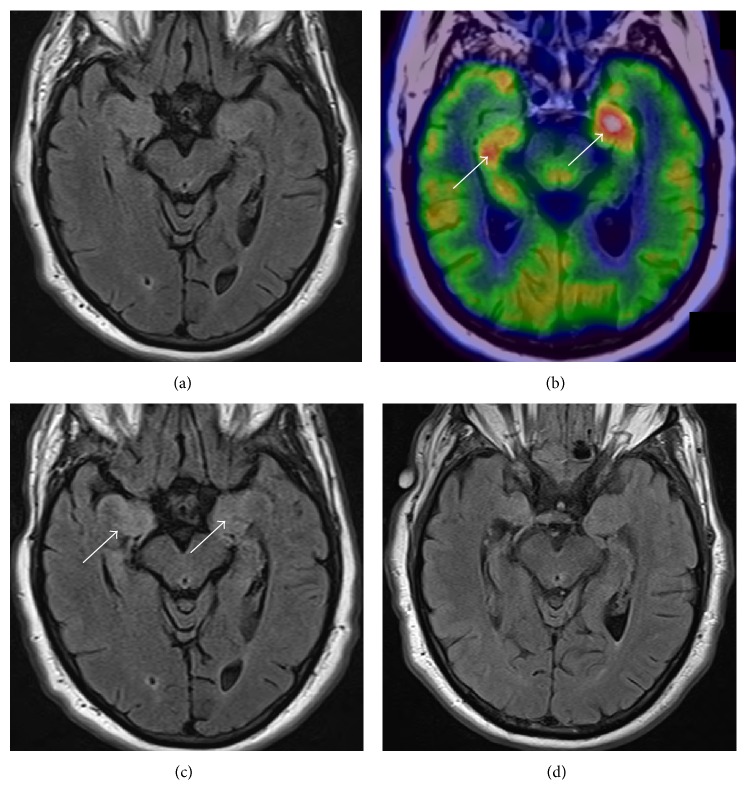
Neuroimaging of VGKC antibody. The initial, unremarkable axial T2/FLAIR MRI of the brain (a) followed by FDG-PET showing significant asymmetric hypermetabolism of the bilateral mesial temporal lobes (arrows; (b)) with repeat axial T2/FLAIR demonstrating hyperintensity in bilateral mesial temporal lobes (arrows; (c)) that significantly improved by the last T2/FLAIR (d).

FDG-PET scans were available in six of the patients. All six patients had hypermetabolism of mesial temporal structures (Table 3). In three of these cases, the FDG-PET was positive (i.e., mesial temporal lobe hypermetabolism) before any changes were visualized on brain MRI. Serial MRI scans in two of the patients subsequently became positive for mesial temporal lobe hyperintensity 1 and 72 days following the FDG-PET scans. These patients were positive for VGKC. The brain MRI for the patient positive for NMDA AE only showed generalized atrophy on subsequent imaging.  Figure 1 is an illustrative example of FDG-PET findings prior to MRI findings. CT of head was unremarkable for any acute process in all the presented cases.

## 4. Discussion

This case series of VGKC and NMDA autoimmune encephalitis illustrates the importance of neuroimaging with emphasis of early FDG-PET particularly in cases of initial nondiagnostic MRIs. It also demonstrates the extralimbic nature that may occur with VGKC antibody positive AE.

It is accepted that paraneoplastic and nonparaneoplastic antibodies can present at distant sites from the limbic system, but they usually do so in addition to limbic involvement [9]. The importance of the solitary finding in this case is not clear. VGKC receptors are known to be found in the peripheral nervous and autonomic nervous systems in addition to the central nervous system. Recent studies have illustrated that antibodies directed to proteins (e.g., contactin-associated protein-like 2 (CASPR2) and leucine-rich glioma-inactivated protein 1 (LGI1)) within the VGKC complex or to Kv1 channels may explain why patients present with such varied clinical and radiological presentations [8, 10, 11]. The diversity in neurologic phenotypes seems to be independent of the VGKC antibody titer level [8]. Even at low levels of positive VGKC, the finding of LGI1 and/or CASPR2 antibodies can result in a diverse spectrum of neurologic phenotypes [8]. Nevertheless, LGI1 antibodies typically result in higher titers of VKGC antibody titers, and LGI1 antibodies will typically have cortical (e.g., seizures and/or altered mental status) presentations [8]. These subtypes of VGKC antibody can be found in patients with extralimbic presentations [8]. This suggests a critical evaluation of extralimbic regions of the brain for MRI changes in patients with suspicion for AE. Importantly, VGKC positivity in the absence of antibodies to either LGI1 or CASPR2 does not seem to be clinically relevant as a marker of autoimmune inflammation [11]. Unfortunately, our seropositive-VGKC patients had serum samples tested prior to the accepted reporting of the subtypes of the VGKC macromolecule complex.

Why VGKC antibodies caused mostly limbic hyperintensity on MRI and the NMDA antibodies did not is not entirely known. It has been suggested that the restricted diffusion seen on the diffusion weighted images (DWI) is due to the presence of abundant number of inflammatory cells along with increased amounts of interstitial water which accounts for the relative normal apparent diffusion coefficient (ADC) images [12]. The hyperintensity is typically asymmetric and rarely enhances with contrast [13]. VGKC are also among the earliest ion channels to appear during brain development. It is not clear whether limbic involvement represents concentration of VGKC in these patients implying a pathogenetic role of the VGKC antibodies or presence of antibodies is an incidental finding. Importantly, the hyperintensity on MRI can persist over months to years and may actually lead to progressive mesial temporal sclerosis [14].

Another important finding in this case series is the lag time in developing noticeable changes on MRI. Two cases showed mesial temporal lobe hypermetabolism on FDG-PET prior to changes on brain MRI. Additionally, the patient with NMDA AE showed mesial temporal lobe hypermetabolism without MRI findings. The utility of FDG-PET in the diagnosis and monitoring of autoimmune encephalitis has been shown to be significant [15–17]. FDG-PET of the brain typically shows hypermetabolism in similar regions as the brain MRI hyperintensity [9]. The described findings on brain MRI have included T2/FLAIR hyperintensities in the mesial temporal lobes (either unilateral or bilateral) that rarely enhance, generalized atrophy, or corpus callosum or basal ganglia hyperintensities [3, 18]. Thus, it is recommended that FDG-PET be obtained early in diagnostic workup of suspected cases of autoimmune encephalitis, especially when the initial MRI of the brain is unremarkable.

In conclusion, FDG-PET is a useful tool in the diagnostic workup of autoimmune encephalitis, such as in patients with VGKC or NMDA receptor antibodies. FDG-PET can be positive despite an unremarkable brain MRI. Additionally, maintaining awareness of extralimbic hyperintensity on MRI is also recommended.

## Figures and Tables

**Table 1:** Patient characteristics.

Patient	Sex	Age (years)	Clinical features	Cancer	Antibody monitoring (days)	VGKC titers (nmol/L)	NMDA serology	Symptom onset to diagnosis (days)	CSF WBC (cells/mm^3^)	CSF protein (mg/dL)	Treatment	Max number of AEDs
1	F	91	Altered mental status	No	Initial	0.13	NA	8	3	57	None	None

2	M	59	Autonomic seizure	No	Initial 54 161 273	1.58 4.22 15.8 1.94	NA	94	1	40	IVMP × 5 days, PLEX × 5 days	3

3	F	52	Altered mental status, depression	No	Initial	0.13	NA	178	NA	69	IVMP × 5 days, IVIG × 5 days	2

4	F	35	Flu-like symptoms, complex partial seizure, hyponatremia, elevated microsomal and thyroglobulin antibodies	No	Initial 24	2.8 0.77	NA	54	2	34	IVIG × 3 days, IVMP × 3 days, PLEX × 5 days	5

5	M	54	Altered mental status, hyponatremia, gelastic seizure	No	Initial	3.89	NA	185	12	47	IVMP × 5 days, PLEX × 5 days	4

6	M	84	Altered mental status, hyponatremia, tonic seizure	No	Initial	0.13	NA	177	2	48	IVMP × 5 days, PLEX × 3 days/week for 2 weeks	2

7	M	22	Psychosis	No	Initial 56	NA	Positive Positive	19	64	23	IVMP × 5, PLEX × 10 d, IVIG × 5, cyclophosphamide, prednisone 5 qd	5

*Average:*		*56.7*			*51.6*	*3.5*		*102.1*	*12.0*	*45.4*		

VGKC, voltage-gated potassium channel antibody; CSF, cerebrospinal fluid; IVMP, 1 gram of intravenous methylprednisolone; IVIG = 0.4 g/kg/d of intravenous immunoglobulin; PLEX, plasma exchange; F, female; M, male; NA, not applicable.

**Table 2:** Structural and physiological workup.

Patient	Days from onset to first image	First image modality	Days from onset to first positive image finding	First positive image modality	Days from onset to first EEG	Days from onset to first positive EEG findings	EEG finding
1	4	CT	NA	NA	159	NA	Normal
2	46	MRI	109	PET	46	81	Vertex seizure
3	73	MRI	120	PET	83	180	Periodic pattern
4	184	MRI	184	MRI	183	183	Nonlocalizable status
5	10	MRI	18	MRI	53	NA	Generalized slowing
6	14	CT	16	MRI	22	22	Right frontotemporal seizure
7	9	CT	33	PET	14	18	Bifrontal seizure

*Average*	*48.6*		*80.0*		*80.0*	*96.8*	

EEG, electroencephalogram; CT, computed tomography; PET, positron emission tomography; NA, not applicable.

**Table 3:** MRI and PET findings.

Patient	MRI brain findings	PET findings
1	Global atrophy out of proportion to patient's age	NA
2	Bilateral mesial temporal lobe hyperintensity on T2/FLAIR without contrast enhancement	Left mesial temporal lobe hypermetabolism
3	Bilateral hyperintensity on T2/FLAIR in the restiform bodies bilaterally without contrast enhancement	Right temporal lobe hypermetabolism
4	Left unilateral mesial temporal lobe hyperintensity on T2/FLAIR without contrast enhancement	Left temporal lobe hypermetabolism
5	Right unilateral mesial temporal lobe hyperintensity on T2/FLAIR without contrast enhancement	Bilateral temporal lobe hypermetabolism
6	Right unilateral mesial temporal lobe hyperintensity on T2/FLAIR without contrast enhancement	Bilateral temporal lobe hypermetabolism
7	Unremarkable brain	Bilateral temporal lobe hypermetabolism
